# Successful Arterial Embolisation of Giant Liver Haemangioma

**DOI:** 10.1155/1993/76519

**Published:** 1993

**Authors:** Yves Panis, Pierre-Louis Fagniez, Daniel Cherqui, Alain Roche, Jean-Claude Schaal, Daniel Jaeck

**Affiliations:** 1 Service de Chirurgie Digestive Hôpital Henri-Mondor Créteil France; 2 Service de radiologie Institut Gustave Roussy Villejuif France; 3 Service de Chirurgie Générale Hôpital Hautepierre Strasbourg France; 4 Service de Chirurgie Digestive hôpital Henri-Mondor 51 av. du Maréchal de Lattre-de-Tassigny Créteil 94000 France

## Abstract

A 28-year old man presented with a symptomatic giant haemangioma. On June 26, 1983, at laparotomy, no resection was attempted because the lesion involved the right lobe of the liver and a part of segments II and III. The patient underwent a right hepatic arterial embolisation with gelatine sponge particles. During follow-up, the patient remained asymptomatic. Five-year review by CT-scan showed a diminution of the size of the haemangioma and hypertrophy of the left lobe. On October 21, 1988, the patient was reoperated on for liver abscess and complete necrosis of the haemangioma. A right hepatectomy was performed. In conclusion, the long-term effect of hepatic arterial embolisation, as demonstrated in our case by regular CT-scans, is useful in cases of diffuse haemangioma as an alternative to hazardous major liver resection. To our knowledge, the long-term effect of hepatic arterial embolisation on symptoms and tumor size have never been reported for giant liver haemangioma.


*HPB Surgery*, 1993, Vol. 7, pp. 141–146
Reprints available directly from the publisher
Photocopying permitted by license only

© 1993 Harwood Academic Publishers GmbH
Printed in the United States of America

### CASE REPORT

### SUCCESSFUL ARTERIAL EMBOLISATION OF GIANT LIVER HAEMANGIOMA

### Report of a case with five-year computed tomography follow-up

YVES PANIS[1], PIERRE-LOUIS FAGNIEZ[1], DANIEL CHERQUI[1], ALAIN ROCHE[2], JEAN-CLAUDE SCHAAL[3] and DANIEL JAECK[3]
[1]*Service de Chirurgie Digestive, Hôpital Henri-Mondor, Créteil, France*
[2]*Service de radiologie, Institut Gustave Roussy, Villejuif, France*
[3]*Service de Chirurgie Générale, Hôpital Hautepierre, Strasbourg, France*

*(Received 8 February 199*

A 28-year old man presented with a symptomatic giant haemangioma. On June 26, 1983, at laparotomy, no resection was attempted because the lesion involved the right lobe of the liver and a part of segments II and III. The patient underwent a right hepatic arterial embolisation with gelatine sponge particles. During follow-up, the patient remained asymptomatic. Five-year review by CT-scan showed a diminution of the size of the haemangioma and hypertrophy of the left lobe. On October 21, 1988, the patient was reoperated on for liver abscess and complete necrosis of the haemangioma. A right hepatectomy was performed. In conclusion, the long-term effect of hepatic arterial embolisation, as demonstrated in our case by regular CT-scans, is useful in cases of diffuse haemangioma as an alternative to hazardous major liver resection. To our knowledge, the long-term effect of hepatic arterial embolisation on symptoms and tumor size have never been reported for giant liver haemangioma.

KEY WORDS: Liver haemangioma, arterial embolisation, hepatectomy

Hepatic haemangioma is the most commonly detected benign liver tumor[1]. It is now well established that small liver haemangiomas (< 4 cm) are largely asymptomatic and can be ignored without any risk of complications or missing malignant lesions[1]. On the other hand, large haemangiomas are more often symptomatic and present atypical ultrasound findings[1,2]. According to most of the authors, surgical resection is indicated in large and symptomatic haemangiomas[3]. In the case of large haemangiomas, ligation of the hepatic artery or radiation therapy have been proposed but with controversial results and have now been abandoned by several authors[3]. Few cases of hepatic arterial embolisation have been reported for giant haemangioma[4,5] but with little effect on symptoms[4] and no strong evidence of a real long-term effect[6].

Address correspondence to: Professor Pierre-Louis Fagniez, Service de Chirurgie Digestive, hôpital Henri-Mondor, 51 av. du Maréchal de Lattre-de-Tassigny, 94000, Créteil, France. (Tél. number: 49812430; Fax number: 49812432).

We present herein a patient with a giant haemangioma treated successfully by arterial embolisation, as demonstrated by a 5-year computed tomography (CT-scan) follow-up.

### CASE REPORT

A 28-year old man presented with a two months history of right upper quadrant pain, without other symptoms. At physical examination, the liver was enlarged with a smooth surface. The ultrasound showed a large heterogeneous tumour with hyperechoic pattern, involving the right lobe of the liver; angiography showed a large hypervascular lesion, without arterioportal fistula; there was no portal vein thrombosis; CT-scan showed a low-density, heterogeneous mass (17.5 × 16 cm), with areas of lower density and calcification (Figure 1), compressing the median hepatic vein; liver tests and serum alphafoetoprotein level were normal. A definite preoperative diagnosis could not be made.

On June 26, 1983, the patient was operated on both for diagnosis and resection: a typical giant cavernous haemangioma involving setments IV to VII and a part of segments II and III was found; it was considered that a major liver resection, outweighed the risk of the haemangioma itself and for this reason, no resection was performed; the extratumourous liver was considered normal. No biopsy of the tumor was performed.

On June 29, 1983, the patient underwent a right hepatic arterial embolisation with gelatine sponge particles (Spongel°); angiography repeated immediately after the embolisation procedure showed complete occlusion of the right branch of the hepatic artery. Prophylactic treatment with metronidazole (Flagyl°) was given for 5

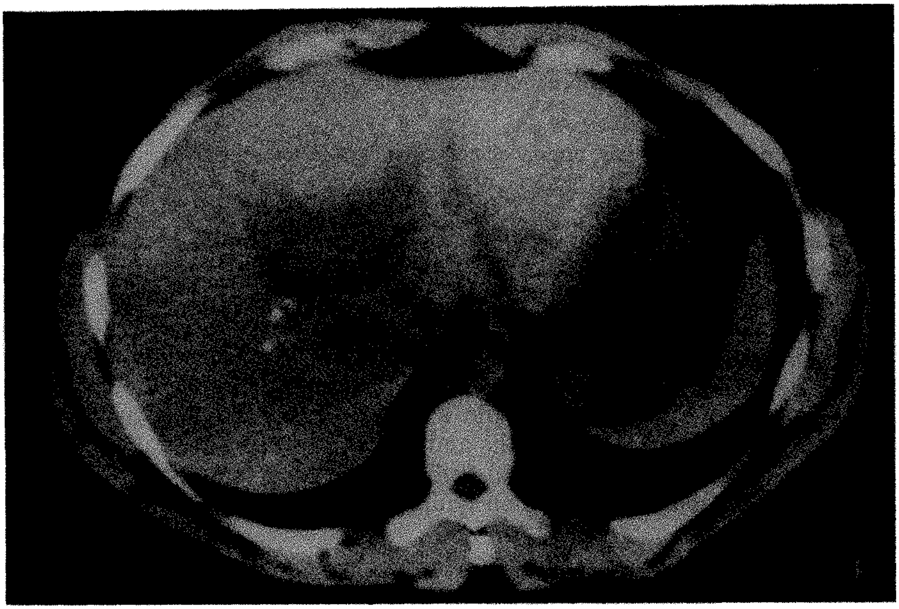

**Figure 1** Preoperative CT-scan showing a low-density, heterogeneous mass (17.5 × 16 cm), with areas of lower density and calcifications.

days. After embolisation, the patient experienced fever (up to 40° C) for 17 jours, with positive blood cultures (Staphylococcus aureus) successfully treated by parenteral antibiotics for 10 days (gentalline, penicillin and metronidazole); at day 1 post-embolisation, amintotransferases levels were raised to 500 IU/L (normal value < 20 IU/L), peaking at 1490 IU/L on day 3, but returned to the normal level on day 10.

On July 12, 1983 (day 13 post embolisation) CT showed multiple oxygen embolism areas within the right lobe of the liver (Figure 2).

During follow-up, the patient remained clinically and biologically asymptomatic. Regular control by CT-scan showed: (a) a diminution of the size of the haemangioma, from 17.5 to 12 cm for the largest diameter; (b) hypertrophy of the left lobe of the liver; (c) a progressive disappearance of oxygen embolism areas; (d) a modification of the density of the lesion which progressively became completely hypodense. On September 2, 1988, five years after embolisation, CT showed a hypodense lesion of the right liver (largest diameter 12 cm), with peripheric calcifications (Figure 3). On October 21, 1988, the patient was reoperated on for recurrent right upper quadrant pain with fever and chills, suggesting infection of the lesion; percutaneous drainage was not attempted because of the heterogenicity and suspected high viscosity of the abcess on CT-scan; a right hepatectomy was performed; pathologic examination confirmed the diagnosis of liver abscess with complete necrosis of the haemangioma. The postoperative course was uneventful. Postoperative CT-scan on February 12, 1989 showed hypertrophy of the left liver remnant, without any focal lesion. Three years after operation, the patient is asymptomatic (with normal liver function tests).

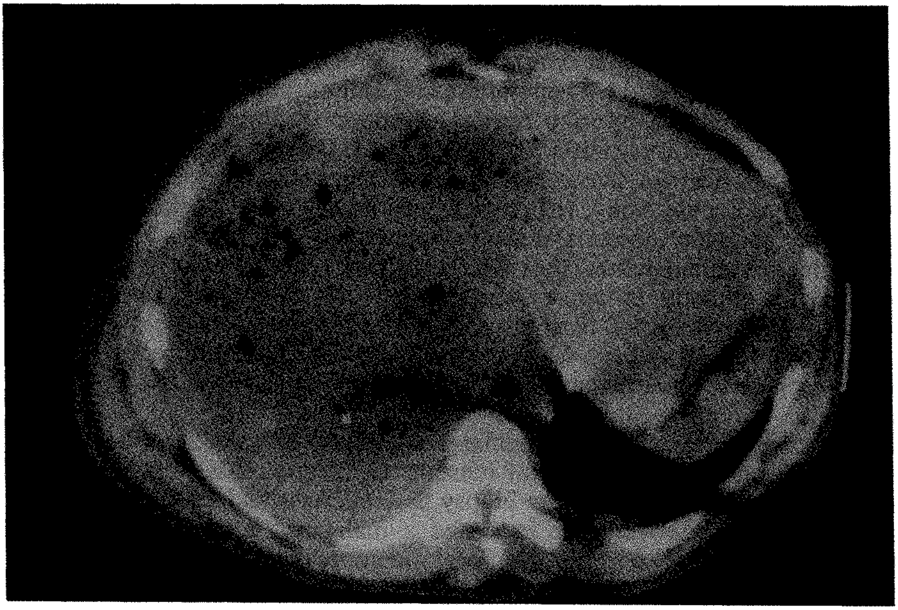

**Figure 2** CT-scan 13 days after embolisation showing multiple oxygen embolism areas within the right lobe of the liver.

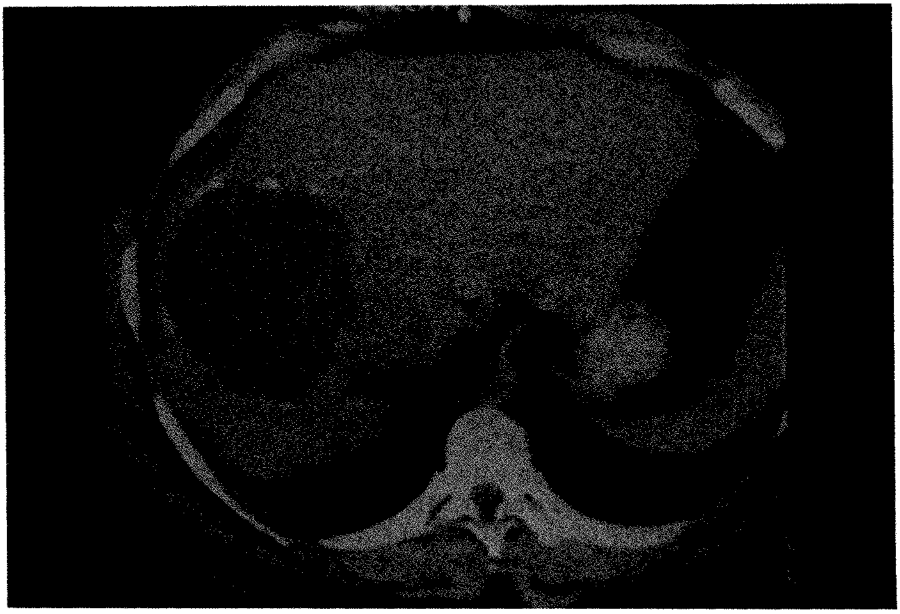

**Figure 3** CT-scan five years after embolisation showing a hypodense lesion of the right liver (largest diameter 12 cm), with peripheric calcifications.

### DISCUSSION

This report demonstrates that symptomatic unresectable giant liver haemangioma can be successfully treated by hepatic arterial embolisation. The rationale of this therapeutic strategy is supported by: (a) complete disappearance of right upper quadrant pain after embolisation; (b) sequential CT-scan follow-up, clearly showing a long-term effect for five years; (c) significant reduction of the lesion's size, permitting right hepatectomy; (d) pathological examination of the resected specimen showing complete necrosis of the haemangioma.

Giant liver haemangioma, those larger than 4 cm, remain silent and are discovered incidentally in approximately 50% of the cases[1]; association with abdominal pain[1,2], abnormal liver tests[2], acute inflammatory process[7] or consumptive coagulopathy (Kasabach-Merritt syndrome)[8] have been reported. In our patient, despite a voluminous lesion greater than 17 cm in diameter, only pain was noted.

In cases of symptomatic giant haemangioma, surgical resection has been advocated by most authors[3,6] for several reasons: (a) preoperative diagnosis remains difficult to obtain, a percutaneous biopsy is dangerous and heterogenicity of the tumour on imaging procedures[1] can miss a malignant lesion; (b) spontaneous intraperitoneal rupture and intratumoral haemorrhage have been reported[9,10] especially in haemangioma greater than 10 cm in diamter; (c) controversial results have been reported with alternative procedures such as hepatic artery ligation or radiotherapy with no long-term effect demonstrated and the potential risk of veno-occlusive disease after radiotherapy[11,12]; (d) series of surgical resection of giant haemangioma without mortality and significant morbidity have recently been reported[2,3,7,8,13].

Hepatic arterial embolisation is, after surgical resection, the most widely reported method for treatment of hepatic haemangioma[6]. Controversial results, especially for symptoms have been reported[4,14]. Significant complications have been reported (i.e. multiple hepatic abscess)[9]. For these reasons, most authors used hepatic arterial embolisation only for irresectable lesions[7]. Usually, as in our patient, multiple hypodense areas within the embolised lobe of the liver are observed on CT-scan. It seems to be due to oxygen released from oxyhaemoglobin trapped in blood cells in the embolized vessels rather than to gas from anaerobic infection[5].

In our patient, alternative treatment as ex-vivo liver surgery could be theoretically proposed: this procedure has recently permitted major liver resection, even for benign liver lesions[15]. However, our case demonstrates that even for very large haemangioma, hepatic arterial embolisation can be considered as a curative treatment. Regular CT-scan control allowed the principal complication of the treatment to be detected (i.e. abscess formation in necrotic lesions), prior to the usual treatment by percutaneous drainage[16]. In our patient, right hepatectomy was performed because of the heterogenicity and suspected high viscosity of the abscess on CT-scan, precluding efficient percutaneous drainage under CT-scan guidance[17].

To our knowledge, the long-term effect of hepatic arterial embolisation on symptoms and tumor size have never been reported. CT-scan follow-up in our patient clearly showed the efficacy of hepatic arterial embolisation on tumor size. In addition, it allowed a safe right hepatectomy to be performed five years later. In recent reports of symptomatic giant haemangiomas, no alternative treatment to surgical resection was proposed, even in the case of major liver resection[18,19].

In conclusion, long-term effect of hepatic arterial embolisation, as demonstrated in our case by regular CT-scan, supports its use in the case of large haemangioma as an alternative to hazardous major liver resection. Then, complete treatment of an haemangioma can be achieved by a conservative approach. This should be preferred to hepatic artery ligation or radiotherapy.

### *References*

1. Gandolfi, L., Leo, P., Solmi, L. *et al.* (1991) Natural history of hepatic haemangiomas: clinical and ultrasound study. *Gut*, **32**, 677–680
2. Pateron, D., Babany, G., Belghiti, J. *et al.* (1991) Giant haemangioma of the liver with pain, fever, and abnormal liver tests. *Dig.Dis.Sci.*, **36**, 524–527
3. Iwatsuki, S., Todo, S. and Starzl, T. (1990) excisional therapy for benign hepatic lesions. *Surg.Gynecol.Obstet.*, **171**, 240–246
4. Martin, B., Roche, A. and Radice, L. (1986) L'embolisation artérielle a t-elle une place dans le traitement des hémangiomes caverneux du foie de l'adulte? *Presse Med.*, **15** 1073–1076
5. Allison, D.J., Jordan, H. and Hennessy, O. (1985) Therapeutic embolisation of the hepatic artery: a review of 75 procedures. *Lancet*, **i**, 595–598
6. Hobbs, K.E.F. (1990) Hepatic hemangiomas. *World J.Surg.*, **14**, 468–471
7. Bornman, P.C., Terblanche, J., Blumgart, R.L., Harries Jones, E.P. and Kalvaria, I. (1987) Giant hepatic haemangiomas: diagnostic and therapeutic dilemmas. *Surgery*, **101**, 445–449
8. Kawarada, Y. and Mizumoto, R. (1984) Surgical treatment of giant haemangioma of the liver. *Am.J.Surg.*, **148**, 287–291
9. Reading, N.G., Forbes, A., Nunnerley, H.B. and Williams, R. (1988) Hepatic haemangioma: a critical review of diagnosis and management. *Q.J.Med.*, **67**, 431–445
10. Sewell, J.H. and Weiss, K. (1961) Spontaneous rupture of haemangioma of the liver: a review of the literature and presentation of illustrative case. *Arch.Surg.*, **83**, 729–733

11. Nishida, O., Satoh, N., Alam, A.S. and Uchino, J. (1988) The effect of hepatic artery ligation for irresectable cavernous hemangioma of the liver. *Am.Surg.*, **54**, 483–489
12. Okasaki, N., Yoshino, M., Yoshida, T., Ohno, T., Kitagawa, T. and Hattorri, N. (1977) Radiotherapy of hemangioma cavernosum of the liver. *Gastroenterology*, **73** , 353–356
13. Yamagata, M., Kanematsu, T., Matsumata, T., Utsunomiya, T., Ikeda, Y. and Sugimachi, K. (1991) Management of haemangioma of the liver: comparison of results between surgery and observation. *Br.J.Surg.*, **78**, 1223–1225
14. Burrows, P.E., Rosenberg, H.C. and Chuang, S. (1985) Diffuse hepatic hemangiomas: percutaneous transcatheter embolization with detachable silicone balloons. *Radiology.*, **156**, 85–88
15. Pichlmayr, R., Grosse, H., Hauss, J., Gubernatis, G., Lamesch, P. and Bretschneider, H.J. (1990) Technique and preliminary results of extracorporeal liver surgery (bench procedure) and of surgery on the *in situ* perfused liver. *Br.J.Surg.*, **77**, 21–26
16. Wong, K.P. (1990) Percutaneous drainage of pyogenic liver abscesses. *World J.Surg.*, **14**, 492–497
17. Helenon, O., Kracht, M., Mathieu, D., Vasile, N., Rotman, N. and Fagniez, P-L. (1989) Percutaneous management of pyogenic liver abcesses. *HPB Surgery*, **1**, 329–335
18. Lise, M., Feltrin, G., Da Pian, P. *et al.* (1992) Giant cavernous hemangiomas: diagnosis and surgical strategies. *World J.Surg.*, **16**, 516–520
19. Belli, L., De Carlis, L., Beati, C., Rondinara, G., Sansalone, V. and Brambilla, G. (1992) Surgical treatment of symptomatic giants hemangiomas of the liver. *Surg.Gynecol.Obstet.*, **174**, 474–478

*(Accepted by S. Bengmark 21 January 1993)*

